# Supplemental Interscalene Blockade to General Anesthesia for Shoulder Arthroscopy: Effects on Fast Track Capability, Analgesic Quality, and Lung Function

**DOI:** 10.1155/2015/325012

**Published:** 2015-04-29

**Authors:** Martin Zoremba, Thomas Kratz, Frank Dette, Hinnerk Wulf, Thorsten Steinfeldt, Thomas Wiesmann

**Affiliations:** ^1^Department of Anesthesia and Intensive Care Medicine, University Hospital Marburg, 35033 Marburg, Germany; ^2^Department of Anesthesia and Intensive Care Medicine, Clinique Bénigne Joly, 21240 Talant, France; ^3^Department of Anesthesiology, University Medical Center of the Johannes Gutenberg-University Mainz, 55131 Mainz, Germany

## Abstract

*Background*. After shoulder surgery performed in patients with interscalene nerve block (without general anesthesia), fast track capability and postoperative pain management in the PACU are improved compared with general anesthesia alone. However, it is not known if these evidence-based benefits still exist when the interscalene block is combined with general anesthesia.* Methods*. We retrospectively analyzed a prospective cohort data set of 159 patients undergoing shoulder arthroscopy with general anesthesia alone (*n* = 60) or combined with an interscalene nerve block catheter (*n* = 99) for fast track capability time. Moreover, comparisons were made for VAS scores, analgesic consumption in the PACU, pain management, and lung function measurements. *Results*. The groups did not differ in mean time to fast track capability (22 versus 22 min). Opioid consumption in PACU was significantly less in the interscalene group, who had significantly better VAS scores during PACU stay. Patients receiving interscalene blockade had a significantly impaired lung function postoperatively, although this did not affect postoperative recovery and had no impact on PACU times. *Conclusion*. The addition of interscalene block to general anesthesia for shoulder arthroscopy did not enhance fast track capability. Pain management and VAS scores were improved in the interscalene nerve block group.

## 1. Introduction

Interscalene brachial plexus blocks are commonly performed in shoulder surgery and are often combined with general anesthesia for optimal patient comfort. Patients receiving a block for shoulder surgery have reduced postoperative opioid consumption [1–3], early mobilization is easier, and hospital discharge is quicker [4]. Interscalene block alone or combined with intraoperative sedation (but not general anesthesia) is superior to general anesthesia alone, allowing improved fast track capability, better pain management, and earlier hospital discharge [5, 6].

However, in clinical routine many anesthetists and orthopedic surgeons prefer interscalene nerve block (as single-shot or catheter techniques) combined with general anesthesia for arthroscopic shoulder surgery [7]. Interestingly, the impact of this combined strategy on fast track capability and early postoperative course has not been widely investigated, even though the interscalene blockade often results in phrenic nerve palsy [8–10], which affects lung function. Conceivably therefore interscalene block combined with the known negative effects of general anesthesia might not be beneficial. On the other hand, the combination should result in lower intraoperative and PACU opioid consumption and overall better pain control. Thus, postoperative fast track capability might be improved even when the interscalene blockade is combined with a general anesthesia.

Our analysis of patients evaluates the clinical impact of a supplemental continuous interscalene nerve block (CINB) for arthroscopic shoulder surgery in a routine setting. We assumed that a supplemental CINB results in enhanced fast track capability compared with the standard group. This analysis includes fast track capability (≥12 points according to the score of White and Song [11]) as the primary outcome parameter as well as pain scores, analgesic consumption, and lung function in the early postoperative period after general anesthesia.

## 2. Methods

We retrospectively analyzed an unpublished data set subgroup of patients who underwent general anesthesia for all types of surgery (e.g., urology, orthopedic surgery, and general surgery) and were nursed in our PACU between January 31, 2010, and December 31, 2012. Ethics Committee approval (University of Marburg, ref. number AZ 176/09; German Clinical Trial Register DRKS-ID DRKS00006032) was obtained by local authorities for prospective data acquisition (intraoperatively and in the PACU) as well as performance of lung function measurements and other testing in 801 patients who were planned for postoperative PACU surveillance. No active randomization was performed in this patient population. We retrospectively analyzed a subgroup of 159 patients of this data set who underwent arthroscopic shoulder surgery under general anesthesia (GA) or under general anesthesia and CINB (a supplemental continuous interscalene nerve blockade) according to their personal choice.

### 2.1. Regional and General Anesthesia

Twelve hours before surgery, patients were premedicated with oral clorazepate (20 mg). Standard monitoring according to national guidelines was established in the anesthesia induction room. If applicable, an interscalene catheter was applied before induction of anesthesia according to our local standard operating procedures. In brief, the block was performed using an insulated needle and stimulation catheter (19 G Tuohy needle and 20 G catheter, StimuCath, Teleflex, Germany) at stimulation settings of 0.1 ms and 1 Hz in addition to real-time ultrasound visualization (dual-guidance technique). A typical contraction of the biceps muscle up to a stimulation of 0.3 mA at 0.1 ms pulse duration had to be achieved before the local anesthetic was injected via the fixed catheter. 10 cc of ropivacaine (7.5 mg/mL) was initially injected followed by a continuous infusion rate of 6 mL/h ropivacaine (2 mg/mL). General anesthesia was then induced according to our standard operating procedure using fentanyl 3 *µ*g kg^−1^, propofol 1.5–2.5 mg kg^−1^, and a single dose of rocuronium (0.5 mg kg^−1^ ideal body weight) to facilitate intubation. No additional neuromuscular blocking agent was given during surgery. Respiratory settings were standardized and ventilation was adjusted to maintain an end-tidal CO_2_ pressure of approximately 4–4.7 kPa. A maximum peak pressure of 30 cmH_2_O was allowed for pressure-controlled ventilation with tidal volumes of 6–8 mL kg^−1^. The inspiration to expiration ratio was 1 : 1.5 and a positive end expiratory pressure of 8 cmH_2_O was applied throughout in both groups, using an adjusted FiO_2_ of 0.5 during anesthesia maintenance and a FiO_2_ of 1.0 before extubation. To monitor adequate anesthetic depth levels, self-adhesive BIS –EEG electrode strips (BIS Quattro; Aspect Medical Systems, Freising, Germany) were positioned on the forehead as recommended by the manufacturer. Anesthesia was maintained by sevoflurane (0.5–2 Vol%) and intermittent bolus application of fentanyl 0.5–2 *µ*g kg^−1^ to maintain BIS within a range of 40–60. The TOF ratio was controlled (TOF-Watch, Organon Teknika, Eppelheim, Germany) to ensure a ratio >0.90 before extubation. A warming blanket (Bair Hugger, Arizant, Trittau, Germany) was applied during surgery. Each patient received dexamethasone (4 mg i.v. after induction) and granisetron (1 mg i.v. 15 min before extubation) as prophylaxis against nausea and vomiting. All patients received standardized basic intravenous (i.v.) nonopioid analgesia (metamizole 15–25 mg kg^−1^) 15 minutes before the estimated end of surgery. When the patients were fully awake and breathing spontaneously, extubation was performed with a FiO_2_ of 1,0. They were then transported to the postanesthesia care unit (PACU) where they were nursed in the half-sitting, head-up position.

### 2.2. Evaluation of Fast Track Score and Postoperative Management

PACU nurses performed postoperative surveillance and nursing according to our local guidelines. Research staff members observed and assessed the fast track criteria (see Table 5) according to White and Song [11] for each patient after arrival at PACU at the given time points. Patients were deemed eligible for discharge from PACU if 12 or more of 14 scoring points were achieved. All patients of the quality control study were nursed in the PACU at least until the measurements at T2 h were successfully performed in the PACU. VAS scales were evaluated at 15-minute intervals. According to our local guidelines, the opioid piritramide i.v. was given as a bolus of 3.75 mg whenever the visual analogue scale (VAS) was >4. Antiemetic drugs were given according to our local guidelines.

### 2.3. Spirometry and Pulse Oximetry

Spirometry and pulse oximetry were standardized as described before [12, 13]. In brief, spirometry measurements were performed with each patient in a 30° head-up position after breathing room air without supplemental oxygen for 5 minutes. Baseline spirometry and pulse oximetry were performed at the preanesthetic visit after thorough demonstration of the correct method. We measured forced vital capacity (FVC), forced expiratory volume in 1 s (FEV1), midexpiratory flow (MEF25–75), and peak expiratory flow (PEF), according to the criteria of the European Respiratory Society [14]. In the PACU, spirometry was performed (T0 h) as soon as the patient was alert and cooperative (mandatory fast track score >10; pain and dyspnea were assessed during coughing before and, if necessary, after analgesic therapy). Spirometry was repeated in the PACU by research staff members at 30 min (T0.5 h) and 2 h (T2 h). Analgesic requirements were documented before each assessment and as soon as the patients were pain-free during coughing.

Patients were discharged after the final evaluations in the PACU at T2 h to the respective wards. Pain management including nerve block catheter techniques in the CINB group was delivered according to local standards. Catheters were regularly removed at the 2nd or 3rd postoperative day.

### 2.4. Statistics

In this retrospective analysis, post hoc power analysis performed with the G^*^ Power software (G^*^Power, Release 3.1, Faul et al. [15], Düsseldorf, Germany) suggested that the numbers of 59 (GA group) and 99 (CINB group) patients per group should provide a power (1-*β*) of >95% to detect an absolute difference of time to fast track capability score ≥12 of 10 min (e.g., 20 versus 30 min in both groups) with an expected standard deviation (SD) of 15 min in both groups with an *α*-error of 5% (*p* < 0.05). To compare differences in postoperative convalescence time, we tested our main hypothesis that time to fast track score ≥12 was significantly lower in the CINB group compared with the GA group.

Moreover, statistical analysis of secondary endpoints was performed for analgesic drug consumption, VAS levels, and lung function. *t*-test or Chi-square testing was performed at each time point at a level of *p* < 0.05 when appropriate. Statistical analysis of spirometry data was performed with relative changes compared to corresponding preoperative baseline values of each group as described before [12]. Statistical analysis was realized with JMP 8 for Windows (SAS Institute Inc., Cary, NC).

## 3. Results

**Figure 1:**
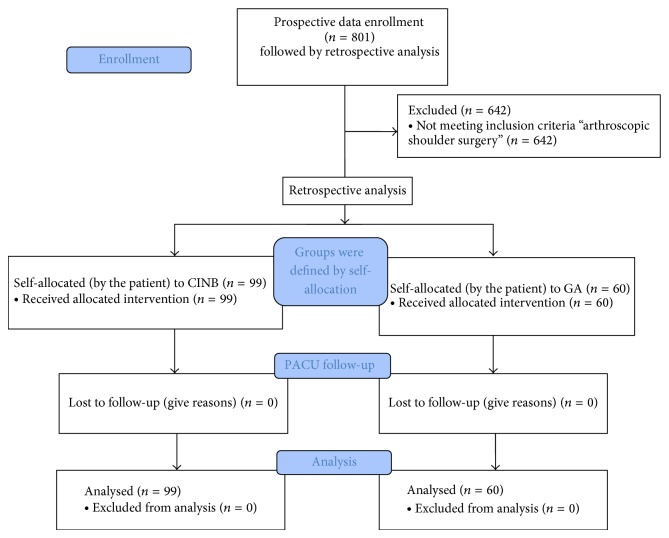


Within the prospectively collected data sets of 801 patients, 159 patients were scheduled for shoulder arthroscopy, of whom 99 received an interscalene nerve block catheter followed by general anesthesia (GA), and 60 had only general anesthetic (see flow diagram in Figure 1). Retrospective analysis was performed using the data sets of this patient subpopulation. No patient scheduled for shoulder arthroscopy was excluded from the analysis.

### 3.1. Preoperative and Intraoperative Data

There were no significant preoperative differences between the groups (Table 1). There were no differences in the level of anesthesia according to BIS (Table 2). However, GA only patients required statistically significant more fentanyl during operation than CINB patients (0.45 mg versus 0.38 mg, *p* = 0.0058).

### 3.2. PACU Discharge and Postoperative Pain Management

With regard to the primary objective, PACU discharge criteria (fast track score >12) were reached within similar times (22 ± 19 min versus 22 ± 18 min; Table 3) in both groups. There were no differences in preoperative VAS scores (Table 1). In the CINB group, VAS scores at PACU arrival (T0) and T0.5 h were significantly less, but there were no differences at T1 h and T2 h. Opioid consumption (piritramide) in the CINB group was less. No postoperative nausea or vomiting was observed.

### 3.3. Spirometry and Pulse Oximetry

Preoperative measurements were within the normal range and did not differ between groups (Table 4). During PACU stay, the CINB group displayed significantly worse FVC, FEV1, PEF, and MEF25–75 measurements than the GA alone group. There were no differences in pulse oximetry between groups at any time point (Table 3). None of the patients complained about dyspnea during PACU stay.

## 4. Discussion

Our retrospective data analysis shows no significant differences between groups regarding their fast track capability (modified fast track score > 12) or VAS scores at T1 and T2. However, intra- and postoperative opioid administration was significantly less in the CINB group resembling better pain control in this group compared with GA alone. In addition, the mean time to reach a fast track score > 12 was reached within 22 min in both groups resembling fast postoperative recovery. Postoperative lung function measurements were significantly worse in the CINB group than the GA group compared with the respective preoperative baseline measurements. However, pulse oximetry revealed no differences between groups. In addition, none of the patients complained about subjective dyspnea.

Interscalene nerve blocks (as single-shot or continuous techniques) are widely used to improve postoperative pain management [6, 16]. Patients receiving nerve blocks without general anesthesia recover more quickly than those given general anesthesia [1]. This strategy is also cost-effective, compared with GA alone [17]. Nevertheless, peripheral regional anesthesia is often combined with GA for better patient comfort or due to the needs of the surgeon (e.g., neuromuscular blockade, blood pressure control) [18]. Contrary to the positive evidence of multitude of studies comparing interscalene blockade alone versus general anesthesia, only one study investigated early postoperative course in patients undergoing open shoulder surgery in general anesthesia with and without a supplemental interscalene blockade. Gohl et al. investigated the postoperative course in 43 patients undergoing open shoulder surgery with and without interscalene block (performed according to Winnie with a nerve-stimulator based technique) [19]. They found no significant differences between groups regarding PACU discharge time at a classic Aldrete score of 9. As they have mentioned, this unexpected finding might be explained by several bias factors. No comparisons were made of the overall opioid consumption and end-tidal volatile anesthetic concentrations. There was no antiemetic prophylaxis, and about 20% of patients suffered postoperative nausea and vomiting (PONV) which might have prolonged the PACU stay. Moreover, PACU length was not defined as the time span until the defined Aldrete score had been reached, but rather as when the patient left the PACU, which might have been affected by logistic issues.

In our study, time to PACU discharge was defined as time to achieve a modified fast track score of 12 according to White and Song [11]. We eliminated the above-mentioned potential bias factors by comparing intraoperative anesthetic and opioid consumption. The opioid-sparing effects of the interscalene blockade did not result in a reduction of opioid related side effects in our study, most probably due to double antiemetic prophylaxis resulting in PONV-free patients of both groups during PACU stay. Nevertheless, the interscalene block did not improve PACU discharge time, although it did in other studies [1, 20]. The CINB group showed lower VAS scores only at T0 and T0.5. This effect is explicable by the greater postoperative opioid consumption in the GA only group. The positive effects of interscalene blockade for postoperative pain management are well known [2, 5, 21–23] and are confirmed by our study results.

Interscalene block resulted in significant lung function impairment, almost certainly due to phrenic nerve palsy as described by Urmey [8, 9]. However, this did not result in desaturation or prolonged PACU stay. In the study by Hartrick et al. [24] comparing the effects of different volumes of local anesthetic on phrenic nerve function, no patient had a prolonged PACU stay, although this was not systematically evaluated. Riazi et al. [10] showed significant differences of postoperative pulse oximetry values comparing two groups with 5 or 20 mL of ropivacaine 0.5% for interscalene nerve blockade and combination with general anesthesia. This was explained by the 45% versus 100% incidences of phrenic nerve palsy; there was no data on PACU surveillance times or other outcome parameters. We have shown that such inadvertent (but regularly occurring) phrenic nerve palsy does (measured indirectly as a FVC decrease of >25%) have some effect on postoperative lung function. As in the mentioned studies above, phrenic nerve palsy did not result regularly in clinical symptoms or complications. The CINB patients did not complain about dyspnea. However, as this is a retrospective analysis of ASA I–III patients, these results should not be generalized to a population with severe underlying lung diseases.

Moreover, it should be kept in mind that all patients who underwent general anesthesia show a relevant reduction of lung function parameters. The severity of lung function impairment is greater in abdominal or thoracic surgery than in peripheral extremity surgery. This is explainable by a greater decrease of functional residual capacity (FRC) as well as other lung function parameters due to relevant atelectasis formation.

### 4.1. Limitations

Our results have a potential bias in that they are obtained from a retrospective analysis of a prospectively enrolled patient population undergoing shoulder arthroscopy. The prospectively collected data of the cohort study focus only on the early postoperative PACU period. A group of patients undergoing shoulder arthroscopy in regional blockade only would have been of interest for studying the negative effects of general anesthesia. The main limitation of this retrospective analysis is that the success rate of the blocks was not systematically investigated. However, use of a dual-guidance block technique should have resulted in success rates exceeding 90%. Block success evaluations in regional anesthesia studies are time consuming (repetitive sensible testing of heat and cold, pin prick testing, and motor function measurements every 5 min for at least 60 min) and difficult to establish in prospective cohort evaluations.

Long-term effects on functional results (e.g., shoulder joint mobilization, feasibility to perform physical therapy, and incidences of chronic pain) were not evaluated in our study focusing on early postoperative benefits. Furthermore, additional economic aspects such as length of hospital stay were not evaluated in this retrospective analysis.

As the addition of a CINB to a general anesthesia technique showed some benefits regarding analgesic quality but not fast track capability, we would suggest further prospective trials evaluating concrete “fast track concepts” for shoulder surgery with additional features. First, a laryngeal mask instead of an endotracheal tube might allow NMBA- (neuromuscular blocking agents-) as well as opioid-sparing techniques resulting in improved postoperative respiratory muscle strength. Use of remifentanil in a balanced anesthetic technique or TIVA might further enhance postoperative outcome. Liberal antiemetic prophylaxis as in our analysis lowers PONV incidence with positive effects on fast track capability. However, we did not evaluate the incidence of PONV after PACU discharge, which might be of further interest. Nevertheless, the mean time to reach a fast track score of ≥12 was 22 min in both groups, which represents excellent postoperative recovery performance. Detailed fast track protocols (e.g., with avoidance of NMBA, endotracheal intubation, use of TIVA techniques, or solely sedation) might further improve this outcome parameter.

## 5. Conclusion

In our retrospective analysis of patients undergoing shoulder arthroscopy with general anesthesia with or without a supplemental interscalene nerve block, no effect on postoperative fast track capability was found. However, pain management was better in the CINB group. Spirometry results were significantly worse in the CINB group as a result of phrenic nerve palsy, but this had no effect on oxygen saturation or PACU discharge criteria. Prospective, randomized trials are needed to verify the results as well as evaluate fast track concepts for further improvements in postoperative recovery.

## Figures and Tables

**Table 1:** Demographic and preoperative data.

	CINB (*n* = 99)	GA (*n* = 60)
Age (y)	56 (SD 13)	49 (SD 14)
Sex (male/female)	56m/43f	43m/17f
ASA status 1/2/3	24/58/17	22/30/8
Weight (kg)	83 (SD 16)	83 (SD 14)
Height (cm)	172 cm (SD 10)	175 cm (SD 9)
Body mass index (kg/m^2^)	28 (SD 4)	27 (SD 4)
Neck circumference (cm)	39 (SD 4)	40 (SD 4)
Waist circumference (cm)	99 (SD 14)	97 (SD 14)
Upper-lower jaw distance (cm)	5.0 (SD 0.8)	5.2 (SD 0.9)
Serum-creatinine (mg/dL)	0.9 (SD 0.2)	0,8 (SD 0.2)
SpO2 (%)	97.0 (SD 1.4)	97.3 (SD 1.4)
Clorazepate (mg)	26 (SD 11)	29 (SD 13)

CINB, supplemental continuous interscalene block group and general anesthesia; GA, general anesthesia without nerve block. SpO2, pulse oximetry saturation; VAS, visual analogue scale. Data are mean ± SD or number (*n*). No significant differences were found between groups.

**Table 2:** Intraoperative data.

	CINB	GA	*p* value
Propofol (mg)	175 (SD 43)	187 (SD 42)	n.s.
Fentanyl (mg)	0.38 (SD 0.12)	0.45 (SD 0.14)	0.0058
Rocuronium (mg)	42 (SD 11)	42 (SD 8)	n.s.
Sevoflurane, end of operation (% end-tidal)	1.5 (SD 0.4)	1.6 (SD 0.4)	n.s.
BIS intraoperative	37 (SD 6)	38 (SD 6)	n.s.
BIS end of operation	41 (SD 9)	41 (SD 11)	n.s.
Metamizole (mg)	1594 (SD 410)	1.682 (SD 459)	n.s.

Data are mean ± SD. SpO2, pulse oximetry saturation. HR, heart rate. BIS, bispectral index. g, gram.

**Table 3:** Postoperative data.

	CINB	GA	*p* value
Time (min) to fast track score >12	22 (SD 18)	22 (SD 19)	n.s.
PACU piritramide (mg)	7 (SD 2)	11 (SD 6)	0.0303
≥1 dose of analgesics	19 (19%)	20 (33%)	0.0468
VAS T0 h	1.4 (95%KI 1.6–3.2 )	2.4 (95%KI 1.6–3.2)	0.0384
VAS T0.5 h	1.3 (95%KI 0.8–1.9 )	2.2 (95%KI 1.5–2.8)	0.05
VAS T1 h	1.3 (95%KI 0.8–1.8)	1.8 (95%KI 1.3–2.4)	n.s.
VAS T2 h	1.0 (95%KI 0.6–1.4)	1.5 (95%KI 1.0–2.1)	n.s.
SpO2 T0 h (% of preop)	96.7 (SD 4.2)	97.2 (SD 3.4)	n.s.
SpO2 T0.5 h (% of preop)	97.7 (SD 3.5)	98.0 (SD 2.8)	n.s.
SpO2 T1 h (% of preop)	97.9 (SD 3.3)	98.7 (SD 3.3)	n.s.
SpO2 T2 h (% of preop)	98.3 (SD 3.0)	99.3 (SD 2.2)	n.s.

Data are presented as mean ± SD or *n*, number of patients. Level of significance, *p* < 0.05. PACU, postanesthesia care room. PACU score, fast track score according to White and Song (0–14 points); for detailed description see text.

**Table 4:** Lung function measurements.

	CINB	GA	*p* value
FVC preop % of normal	95 (SD 15)	94 (SD 16)	n.s.
FVC % T0 h	56 (SD 18)	73 (SD 18)	<0.0001
FVC % T0.5 h	61 (SD 17)	80 (SD 16)	<0.0001
FVC % T1 h	64 (SD 18)	78 (SD 19)	<0.0001
FVC % T2 h	67 (SD 17)	85 (SD 17)	<0.0001
Patients with >25% decline in FVC at T0 h (*n*)	63	26	0.0002
Patients with >25% decline in FVC at T0.5 h (*n*)	71	19	<0.0001
Patients with >25% decline in FVC at T1 h (*n*)	67	22	<0.0001
Patients with >25% decline in FVC at T2 h (*n*)	54	11	<0.0001
FEV1 preop % of normal	93 (SD 16)	91 (SD 19)	n.s.
FEV1 % T0 h	54 (SD 18)	67 (SD 18)	<0.0001
FEV1 % T0.5 h	59 (SD 18)	76 (SD 17)	<0.0001
FEV1 % T1 h	63 (SD 19)	79 (SD 22)	<0.0001
FEV1 % T2 h	64 (SD 18)	85 (SD 17)	<0.0001
PEF preop % of normal	87 (SD 25)	82 (SD 22)	n.s.
PEF % T0 h	51 (SD 19)	59 (SD 20)	0.0184
PEF % T0.5 h	55 (SD 20)	67 (SD 20)	0.0002
PEF % T1 h	59 (SD 20)	72 (SD 27)	0.0011
PEF % T2 h	61 (SD 22)	82 (SD 26)	<0.0001
MEF25–75 preop % of normal	90 (SD 19)	86 (SD 22)	n.s.
MEF25–75 T0 h	55 (SD 33)	65 (SD 31)	0.0211
MEF25–75 T0.5 h	58 (SD 23)	74 (SD 25)	<0.0001
MEF25–75 T1 h	61 (SD 25)	79 (SD 28)	<0.0001
MEF25–75 T2 h	63 (SD 24)	85 (SD 22)	<0.0001

Data are presented as absolute values (mean ± standard deviation). Statistical analysis between groups was performed with relative changes compared to corresponding preoperative baseline values. For abbreviations, see Table 2. FVC, forced vital capacity. FEV1, 1-second expiratory volume. PEF, peak expiratory flow. MEF25–75, midexpiratory flow (25–75).

**Table 5:** Fast track criteria (according to White and Song).

Criteria		Score
Level of consciousness	Awake and oriented	2
	Arousable with minimal stimulation	1
	Responsive only to tactile stimulation	0

Physical activity	Able to move all extremities on command	2
	Some weakness in movement on extremities	1
	Unable to voluntarily move extremities	0

Hemodynamic stability	Blood pressure <15% of baseline MAP value	2
	Blood pressure 15–30% of baseline MAP value	1
	Blood pressure >30% below baseline MAP value	0

Respiratory stability	Able to breathe deeply	2
	Tachypnea with good cough	1
	Dyspneic with weak cough	0

Oxygen saturation status	Maintains value >90% on room air	2
	Requires supplemental oxygen	1
	Saturation <90% with supplemental oxygen	0

Postoperative pain assessment	None or mild discomfort	2
	Moderate to severe pain controlled with IV analgesics	1
	Persistent severe pain	0

Postoperative emetic symptoms	None or mild nausea with no active vomiting	2
	Transient vomiting or retching	1
	Persistent moderate to severe nausea or vomiting	0

Total score		**14**

Score criteria according to White and Song, eligibility for PACU discharge is a minimum score of ≥12 points. MAP: mean arterial pressure.
